# Gestational surrogacy for women with recurrent pregnancy loss due to refractory chronic histiocytic intervillositis

**DOI:** 10.1111/1471-0528.17522

**Published:** 2023-05-02

**Authors:** Emily F. Cornish, Claudia A. A. Belardo, Roger Turnell, Thomas McDonnell, David J. Williams

**Affiliations:** ^1^ Department of Maternal and Fetal Medicine, Elizabeth Garrett Anderson Institute for Women's Health University College London London UK; ^2^ Chronic Histiocytic Intervillositis Online Support Forum; ^3^ Division of Maternal‐Fetal Medicine University of Alberta Edmonton Alberta Canada; ^4^ Department of Biochemical Engineering, Faculty of Engineering Science University College London London UK

## 1 INTRODUCTION

Chronic histiocytic intervillositis (CHI) is a rare placental disorder that affects approximately 1 in 600 pregnancies.^1^ It is diagnosed when at least 5% of the intervillous space is occupied by maternal CD68^+^ immune cells (histiocytes) and is often accompanied by massive perivillous fibrin deposition.^2^ CHI is strongly associated with miscarriage (24%), stillbirth (29%), fetal growth restriction (72%) and preterm birth (68%).^2^ It carries a high risk of recurrence in subsequent pregnancies.^1^, ^2^ The aetiology of CHI is unclear, but its association with maternal autoimmunity and its histological similarity to rejected solid organ allografts suggest a maternal immune ‘rejection’ of the placenta.^3^ While maternal immunosuppression reduces the histological severity of CHI and can improve live birth rate,^4^ some patients have refractory disease in which every successive pregnancy is affected. Eventually, some of these women resort to gestational surrogacy. There is, however, only one published case of successful surrogate pregnancy in this context.^5^ Here, we report the outcomes of 17 surrogate pregnancies in which the embryos came from 13 women with recurrent adverse pregnancy outcomes due to CHI.

## 2 METHODS

Women with one or more histologically proven cases of CHI who had subsequently undergone IVF and gestational surrogacy were eligible for inclusion. Eligible women were identified through an international online CHI support group. Pregnancy outcome data from autologous and surrogate pregnancies were collected. Birthweight centiles were calculated using the INTERGROWTH‐21st calculator (http://intergrowth21.ndog.ox.ac.uk/en/ManualEntry). This study was approved by the London Research Ethics Committee (Fulham, 19/LO/0105). All participants provided written informed consent for publication.

## 3 RESULTS

Thirteen women with recurrent CHI participated in the study. These women had carried 54 pregnancies themselves (51 singletons, 3 dichorionic‐diamniotic twins). These pregnancies resulted in high rates of adverse perinatal outcomes (Table 1). Of the nine babies born alive, two died in the neonatal period (2/9, 22%), meaning only 7/54 pregnancies (13%) resulted in surviving children. In 8/54 (15%) pregnancies, the mother received antenatal immunosuppression including one or more of prednisolone, hydroxychloroquine, tacrolimus and intravenous immunoglobulin. Following attempts to carry a pregnancy themselves, all 13 women underwent IVF using their own oocytes and their partner's sperm, followed by embryo transfer into a surrogate mother. This led to 17 successful surrogate conceptions (12 singletons, 5 dichorionic‐diamniotic twins), of which 15/17 (88%) ended in term or near‐term live birth with normal birthweight. The two remaining pregnancies ended in first‐trimester miscarriage, one due to fetal trisomy 21 and the other with no identified cause. There were two failed embryo transfers. None of the surrogate mothers received immunosuppression.

Given the good outcomes of the completed surrogate pregnancies, only 4/17 placentas were sent for histopathological analysis and none showed signs of CHI.

Before undergoing IVF and gestational surrogacy, one woman had a late miscarriage and two early miscarriages due to CHI. She then had two successful surrogate pregnancies. The first was described in the cited article by Reus et al.^5^ but the second has not yet been reported, hence her inclusion in this cohort.

The parents of the fetus with trisomy 21 subsequently underwent further IVF using donor oocytes and had healthy dichorionic‐diamniotic twins delivered at 36 weeks' gestation by a surrogate mother.

**TABLE 1:** Maternal demographics and outcomes of 54 autologous pregnancies compared with 17 surrogate pregnancies from 13 women with recurrent CHI.

Maternal demographics	
Total	13
Age at study participation, years; median (IQR)	42 (40–47)
Body mass index, kg/m^2^; median (IQR)	25.5 (23.7–27.7)
Ethnicity: White	10 (77%)
Ethnicity: Asian	2 (15%)
Ethnicity: mixed White/Asian	1 (8%)
Autoimmune disease	2 (15%) – both Graves' disease

	Autologous pregnancy	Surrogate pregnancy	*P‐*value
Obstetric outcomes^a^			
Total	54	17	
Early miscarriage (<14 weeks)	28 (52%)	2 (12%)	0.004
Late miscarriage (14^+0^ to 23^+6^)	8 (15%)	0	–
Termination of pregnancy for severe FGR	3 (6%)	0	–
Stillbirth (≥24 weeks)	6 (11%)	0	–
Live birth: preterm (<37 weeks)	3 (6%)	5 (29%)^b^	0.016
Live birth: term (≥37 weeks)	6 (11%)	10 (59%)	<0.0001
Neonatal death	2 (4%)	0	–
Surviving child	7 (13%)	15 (88%)	<0.0001
Perinatal outcomes for singleton pregnancies^c^			
Gestational age at delivery (weeks); median (IQR)	15.6 (8.0–26.1)	38.4 (36.4–39.9)	<0.0001
Birthweight (g); median (IQR)	320 (144–1900)	3515 (3101–3660)	<0.0001
Birthweight centile; median (IQR)	0.0 (0.0–26.7)	78.8 (40.5–94.7)	0.002
Perinatal outcomes for twin pregnancies^c^			
Gestational age at delivery (weeks); median (IQR)	11.5 (10.0–21.5)	36.1 (36.0–37.0)	<0.001
Birthweight (g) ; median (IQR)	210 (175–245)	2367 (2268–2499)	0.030
Birthweight centile; median (IQR)	0.0 (0.0–0.0)	22.4 (3.2–37.3)	0.030

*Note*: Birthweight centiles were calculated using the INTERGROWTH‐21st birthweight centile calculator.

FGR, fetal growth restriction; IQR, interquartile range.

^a^ *P*‐values calculated with Fisher's exact test.

^b^ Three of the five preterm births in the surrogate pregnancies were elective deliveries of dichorionic‐diamniotic twins at 36 weeks' gestation. The fourth was a singleton delivered after spontaneous onset of labour at 36 weeks and 5 days. The fifth was a singleton delivered by caesarean section at 33 weeks and 6 days due to maternal pre‐eclampsia.

^c^ *P*‐values calculated with Kolmogorov–Smirnov test (given non‐normal distribution of autologous pregnancy outcome data) using graphpad prism.

## 4 CONCLUSIONS

This study demonstrates that when a surrogate mother carries the embryo of a couple affected by refractory CHI, pregnancy outcomes are normalised. Although surrogacy is not universally available or acceptable, it should therefore be considered as an alternative route to parenthood for these women. Changing the maternal ‘host’ appears to be highly effective in preventing recurrent CHI. This observation reinforces the hypothesis that CHI is driven by an abnormal maternal immune response to the placenta, as opposed to being a primary placental disorder.

## AUTHOR CONTRIBUTIONS

EFC, CAAB and DJW conceived and planned the study. TM and RT contributed to study design. EFC and CAAB collected data. EFC, TM, RT and DJW analysed the data. All authors contributed to writing and editing the article.

## FUNDING INFORMATION

EFC is supported by an MRC Clinical Research Training Fellowship (MR/V028731/1), which funds investigation into the pathogenesis of chronic histiocytic intervillositis. TM is supported by an MRF fellowship (MRF‐057‐0004‐RG‐MCDO‐C0800). DJW is supported by the National Institute for Health Research University College London Hospitals Biomedical Research Centre.

## CONFLICT OF INTEREST STATEMENT

None declared.

## ETHICS APPROVAL

This study was approved by the London Research Ethics Committee (Fulham, 19/LO/0105) on 6 February 2019.

## Data Availability

The data that support the findings of this study are available on request from the corresponding author. The data are not publicly available due to privacy or ethical restrictions.
